# Dopamine Suppresses Synaptic Responses of Fan Cells in the Lateral Entorhinal Cortex to Olfactory Bulb Input in Mice

**DOI:** 10.3389/fncel.2020.00181

**Published:** 2020-06-18

**Authors:** Shaolin Liu

**Affiliations:** Department of Anatomy, Howard University College of Medicine, Washington, DC, United States

**Keywords:** dopamine, synaptic transmission, interneuron, olfactory bulb, excitation, inhibition

## Abstract

The lateral entorhinal cortex (LEC) is involved in odor discrimination, odor-associative multimodal memory, and neurological or neuropsychiatric disorders. It receives direct axonal projections from both olfactory bulb (OB) output neurons and midbrain dopaminergic neurons. However, the cellular targets in LEC receiving direct synaptic input from OB output neuron, the functional characteristics of these synapses, and whether or how dopamine (DA) modulates the OB-LEC pathway remain undetermined. We addressed these questions in the present study by combing optogenetic and electrophysiological approaches with four major findings: (1) selective activation of OB input elicited glutamate-mediated monosynaptic responses in all fan cells, the major output neurons in layer II of the LEC; (2) this excitatory synaptic transmission exhibited robust paired-pulse facilitation (PPF), a presynaptically derived short-term synaptic plasticity; (3) DA dramatically attenuated the strength of the OB input-fan cell synaptic transmission *via* activation of D1 receptors; and (4) DA altered the PPF of this transmission but neither intrinsic properties of postsynaptic neurons nor the kinetic profile of postsynaptic responses, suggesting that presynaptic mechanisms underlie the DA inhibitory actions. This study for the first time demonstrates the FCs in the LEC layer II as the postsynaptic target of direct OB input and characterizes DA modulation of the OB input-fan cell pathway. These findings set the foundation for future studies to examine the synaptic transmission from the OB output neuron axon terminals to other potential cell types in the LEC and to pinpoint the pathophysiological mechanisms underlying olfactory deficits associated with DA-relevant neurological and neuropsychiatric disorders.

## Introduction

The entorhinal cortex (EC) is a key structure of the limbic system as it not only gates input and output of the hippocampus (HPC) but also provides pivotal links between many cortical and subcortical regions (Canto et al., 2008; Kobro-Flatmoen and Witter, 2019). Based on its cytoarchitecture and connective relationship with the HPC, EC is divided into two subareas: medial and lateral entorhinal cortices (MEC and LEC; Canto et al., 2008). While MEC is essential for spatial representation (Fyhn et al., 2004; Witter and Moser, 2006), LEC participates in odor discrimination and integration of odor information into associative multimodal memories (Stäubli et al., 1984; Chapuis et al., 2013; Boisselier et al., 2014; Igarashi et al., 2014). LEC receives direct olfactory input from the olfactory bulb (OB; Heimer, 1968; Haberly and Price, 1977; Beckstead, 1978; Wouterlood and Nederlof, 1983; Boeijinga and Van Groen, 1984; Wouterlood et al., 1985; Van Groen et al., 1987), the first station of synaptic processing olfactory signals where the olfactory sensory neurons synapse with the OB output neurons including mitral cells (MCs) and tufted cells (Lledo et al., 2005; Wilson and Mainen, 2006). Both anatomical and electrophysiological evidence shows that only axons of MCs in the OB reach the LEC (Haberly and Price, 1977; Scott, 1981; Igarashi et al., 2012). MC axons terminate in the superficial lamina of the LEC layer I (Kosel et al., 1981; Wouterlood and Nederlof, 1983; Room et al., 1984; Wouterlood et al., 1985) where they synapse onto the dendrites of principal neurons in layers II and III (Wouterlood and Nederlof, 1983). However, the functional characteristics of these OB-LEC synaptic pathways remain largely unknown. This is mainly due to the lack of experimental approaches to differentiate the afferent fibers in the LEC layer I which also harbors input originating from many cortical and subcortical regions.

Also, LEC receives dopaminergic projections from the midbrain and expresses dopamine (DA) receptors (Loughlin and Fallon, 1984; Charuchinda et al., 1987; Oades and Halliday, 1987; Richfield et al., 1989; Köhler et al., 1991; Weiner et al., 1991), implying dopaminergic modulation of olfactory processing in the LEC. The LEC-projecting dopaminergic neurons are located in the midbrain including ventral tegmental area (VTA) and substantia nigra (Collier and Routtenberg, 1977; Loughlin and Fallon, 1984; Björklund and Dunnett, 2007) where dysfunction of dopaminergic neurons was detected in neurological and neuropsychiatric disorders (Sesack and Carr, 2002; Dauer and Przedborski, 2003; Brichta et al., 2013; Morales and Root, 2014; Nobili et al., 2017). For example, degeneration or hyper activities of VTA dopaminergic neurons occur in the early stage of Parkinson’s disease (PD) and Alzheimer’s disease (AD) or schizophrenia, respectively. Like OB output neuron axons, VTA dopaminergic projections also predominantly terminate in the LEC superficial layers (Loughlin and Fallon, 1984; Oades and Halliday, 1987; Erickson et al., 2000), suggesting dopaminergic modulation of the OB-LEC pathways. Interestingly, the olfactory deficit is a common symptom in PD, AD and schizophrenia patients (Rupp, 2010; Fullard et al., 2017; Murphy, 2019), indicating that understanding the dopaminergic modulation of synaptic processing olfactory signals in the LEC potentially sheds light on the pathophysiology of these neurological and neuropsychiatric disorders. Previous studies reported excitatory or inhibitory DAergic modulation of synaptic responses in principal neurons in the LEC depending on DA concentration (Caruana et al., 2006; Caruana and Chapman, 2008; Glovaci and Chapman, 2015), i.e., a low dose of DA acts on postsynaptic D1 receptors to facilitate synaptic transmission while high dose of DA suppresses synaptic responses in LEC principal neurons by inhibiting presynaptic transmitter release *via* activation of D2 receptors.

Here, we report some preliminary findings of a study characterizing the synaptic transmission from the OB output neuron axon terminals to fan cells in layer II of the LEC and examining the modulatory effects of DA at a low concentration on this synaptic pathway with optogenetic and electrophysiological approaches.

## Materials and Methods

### Animals

Wild-type (C57BL/6J) mice of both sexes at the age of 5 weeks were purchased from Jackson Laboratories. All animals were maintained with a standard 12 h light/dark cycle with *ad libitum* access to food and water. All experimental procedures were carried out following protocols submitted to and approved by the Howard University Institutional Animal Care and Use Committee.

### Channelrhodopsin 2 (ChR2) Expression

**Figure 1:**
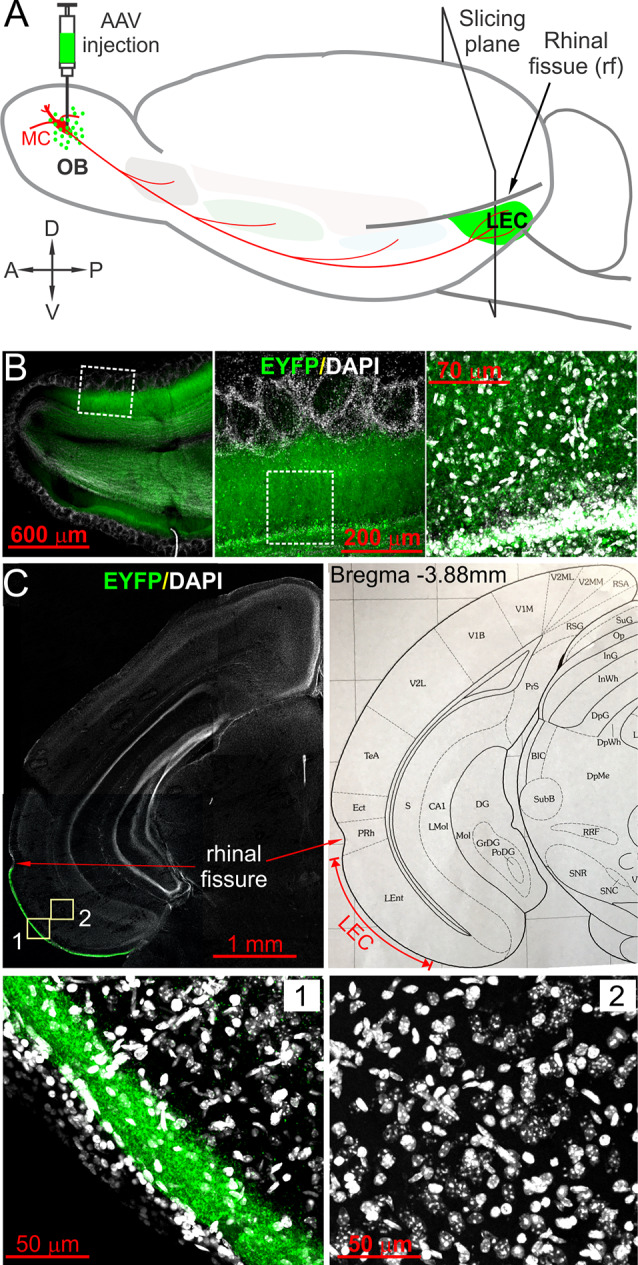
Optogenetic tracing and labeling afferent fibers in the lateral entorhinal cortex (LEC) directly projected from olfactory bulb (OB). **(A)** Schematic showing the experimental design in which AAV5-hSyn-hChR2(H134R)-EYFP (AAV) was injected into the OB. Coronal slices containing LEC were prepared after 4 weeks for ChR2-EYFP expression. **(B)** Left panel showing a confocal photo of a typical horizontal OB slice with ChR2-EYFP expression in local neurons; middle and right panels are blown-up areas highlighting enhanced yellow fluorescent protein (EYFP) expression in the mitral cell and the external plexiform layers. **(C)** Top left panel is a representative confocal photo showing a coronal brain hemispheral slice with selective EYFP expression in layer I of LEC; the top right panel is a corresponding atlas picture illustrating the LEC with the anatomical landmark rhinal fissure. Bottom panels are blown-up photos of the areas correspondingly labeled in the top left panel highlighting EYFP expression selectively in the superficial lamina of layer I of the LEC (1) but no EYFP-expressing somata in the deep layers (2).

Virus injection was performed as previously described (Liu et al., 2013). Briefly, adeno-associated virus serotype 5 (AAV2.5) carrying fusion genes for ChR2 and enhanced yellow fluorescent protein (EYFP) (Tsai et al., 2009; Addgene, Watertown, MA, USA) were injected into the mitral cell layer (MCL) of both medial and lateral sides of each OB between postnatal weeks 5 and 6. Under deep anesthesia with ketamine/xylazine mixture (100 mg/kg ketamine and 10 mg/kg xylazine), the skull was exposed, two craniotomies (0.5 mm diameter) were made over each OB: one on the medial side with typical coordinates at 3.92 mm from Bregma and 0.5 mm from the midline and other on the lateral side with typical coordinates at 3.92 mm from Bregma and 1.25 mm from the midline. AAV2.5 was injected into one point in the MCL on each side with a depth of 1.5 mm at a rate of 0.6 μl/min for 30 s *via* a nanoliter injector (Nanoject III, Drummond Scientific, Broomall, PA, USA). After 4 weeks for ChR2–EYFP expression, acute coronal brain slices containing the LEC were prepared for experiments (Figure 1A).

### Slice Preparation

Acute coronal brain slices containing the LEC were prepared from 6-week old animals deeply anesthetized by isoflurane. Briefly, coronal slices (350 μm) were cut with a VT1200s vibratome (Nussloch, Germany) in an ice-cold and bubbled with carbogen (95% O_2_–5% CO_2_) sucrose-based artificial CSF (sucrose-ACSF) containing (in mM) 210 sucrose, 3 KCl, 1.2 NaH_2_PO_4_, 2.6 MgSO_4_, 0.5 CaCl_2_, 26 NaHCO_3_, 10 glucose. After incubation in normal ACSF at 30°C for 30 min, slices were then transferred to ACSF continuously oxygenated with carbogen at room temperature until they were used for recordings. Normal ACSF consisted of (in mM): 124 NaCl, 3 KCl, 1.25 NaH_2_PO_4_, 2.0 MgSO_4_, 2.0 CaCl_2_, 26 NaHCO_3_, 10 glucose. During experiments, slices were perfused with ACSF equilibrated with carbogen and warmed to 30°C at a flow rate of 3 ml/min.

### Electrophysiology

Whole-cell patch-clamp recordings were made from LEC neurons visualized using Axio Examiner (Zeiss, Oberkochen, Germany) fixed-stage upright microscope equipped with near-infrared differential interference contrast (DIC) optics and an Axiocam 506 color/mono camera (Zeiss, Oberkochen, Germany). Neurons in the LEC were preselected for recording based on their soma location and dendrite projections. Neuron types were subsequently verified by electrophysiological properties and *post hoc* morphology reconstruction.

To visualize the recorded neurons and their dendrites followed by reconstruction, Alexa-594 (10 μM) and biocytin (0.2%) were included in the internal solution. Signals in current or voltage clamp were recorded with a MultiClamp 700B amplifier (Molecular Devices, Palo Alto, CA, USA) and low-pass filtered at 4 kHz and sampled at 10 kHz with a DIGIDATA 1550B 16-bit analog-to-digital converter (Molecular Devices) using Clampex 11.0.3 (Molecular Devices). Recording electrodes (4–7 MΩ) were pulled from standard-wall glass capillary tubes without filament (Sutter Instrument, Novato, CA, USA). Electrode solution contained (in mM) 115 K-gluconate, 5.0 EGTA, 0.63 CaCl_2_, 5.5 MgCl_2_, 10 HEPES, 3 Na_2_-ATP, 0.3 Na_3_-GTP, and 14 Tris-phosphocreatine (pH 7.3, 285–295 mOsm). To record synaptic responses to optical stimulation of OB input, neurons were voltage-clamped at −70 mV, which is close to the average resting membrane potential (−73.4 ± 1.5 mV, *n* = 7) of fan cells.

### Immunochemical Staining

Coronal brain slices with biocytin (0.2%, w/v)-filled neurons were transferred to 4% paraformaldehyde (PFA) immediately after recording and kept at 4°C overnight. Slices were washed three times (5 min each) with 0.05 M phosphate-buffered saline (PBS) before being incubated in a blocker solution on a shaker for 1 h. Blocker solution contains 1% (w/v) bovine serum albumin and 0.5% (v/v) Triton X-100 in 0.05 M PBS. Then the blocker solution was replaced by a fresh blocker solution containing streptavidin-CY3 (1 μg/ml) and the reaction container was covered with aluminum foil to prevent light exposure at room temperature on a shaker for 7 h. To terminate streptavidin-CY3 staining, slices were rinsed with 0.05 M PBS for three times (5 min each) before being treated with 0.05 M PBS containing 4′,6-diamidino-2-phenylindole (DAPI; 5 μg/ml) to stain cell nuclei at room temperature in the dark for 10 min. After three times of wash (5 min each) with PBS to terminate DAPI staining, slices were wet mounted and cover-slipped with fluorescence mounting media. Biocytin-filled cells with CY3 staining were scanned and reconstructed under a confocal microscope.

### Optical Stimulation

Blue light (473 nm) was generated by a diode-pumped and solid-state laser MBL-III-473 (Optoengine) with a maximal power of 100 mW and gated with a laser shutter LST200 (NMLaser Products) or a Polygon 400E (Mightex, Toronto, ON, Canada), a LED illuminator with optical stimulation at the cellular or subcellular resolution. Laser or LED light stimuli were delivered by a 25 μm diameter multimode optical fiber (ThorLabs; 0.1 NA, ~7° beam divergence) or directly through the microscope objective lens, respectively. Onset and duration of laser light stimulation were monitored during every experiment by splitting 1% of the laser beam out to a high speed (30 ns rise-time) silicon photosensor (model 818-BB, Newport) and recorded by the same MultiClamp 700B amplifier.

### Data Analysis

Amplitudes of evoked EPSCs were measured with Clampfit 11.0.3 (Molecular Devices). Drug effects on EPSCs were determined by measuring the amplitudes of 20 traces recorded during a 5 min period immediately before or 10 min after drug application in each condition for each cell. Data were further analyzed and graphed with Origin 2020 (Origin Lab, Northampton, MA, USA). Statistical significance of comparison among average responses of the same group of cells to different treatments was calculated and determined by using ANOVA one-way Repeated Measure with Bonferroni *post hoc* comparisons (for data with more than two repeated treatments) or paired *t-test* in Origin 2020.

### Drugs Delivery and Chemicals

All drugs were bath applied. 6,7-Dinitroquinoxaline-2,3-dione disodium salt (DNQX disodium salt, 10 μM), (S)-(-)-5-Aminosulfonyl-N-[(1-ethyl-2-pyrrolidinyl)methyl]-2-methoxybenzamide [(S)-(-)-Sulpiride, 50 μM], and 8-Bromo-2,3,4,5-tetrahydro-3-methyl-5-phenyl-1H-3-benzazepine-7-ol hydrobromide (SKF83566 hydrobromide, 10 μM) were purchased from Tocris Cookson (Ellisville, MO, USA). Cy3 Streptavidin was from Thermo Fisher Scientific (Waltham, MA, USA). Dopamine chloride (DA, 5 μM) and all other chemicals were purchased from Sigma-Aldrich (St. Louis, MO, USA). All drugs except (S)-(-)-Sulpiride (dissolved in dimethyl sulfoxide) were dissolved in distilled water as stock solution and diluted 1,000 times with ACSF to final concentrations.

## Results

The axonal projections from the OB to the LEC were identified decades ago (Haberly and Price, 1977). Monosynaptic transmission from OB to the LEC was also reported in studies with the extracellular recording (Boeijinga and Van Groen, 1984; Van Groen et al., 1987; Biella and de Curtis, 2000). However, the specific postsynaptic cell types of the OB input in the LEC remain unknown at least partly due to the lack of experimental approaches to selectively activate OB axon fibers, which are intermingled with afferents projected from other brain areas in the LEC layer I (Canto et al., 2008). Here we tried to bridge this gap by combining whole-cell patch-clamp with the optogenetic approach, which enables cell-type-specific activation and long-distance neural fiber tracing (Miesenbock, 2011; Kim et al., 2017).

### Optogenetic Tracing the Pathway From OB to LEC

To selectively label and activate direct OB MC axon fibers to the LEC, we adopted the optogenetic approach by injecting the virus into the OB to express ChR2-EYFP in MCs (Figure 1A), which are the only OB output neurons project to the LEC. Since LEC receives axonal projections not only from the OB MCs but also from output neurons in other OB-targeted brain regions including the piriform cortex and amygdala (Canto et al., 2008), we injected AAV5-hSyn-hChR2(H134R)-EYFP into the MCL on both medial and lateral sides of each OB because AAV5 can be anterogradely transported to axon terminals but does not across synapses to postsynaptic neurons thus only labels direct projections from the OB (Zingg et al., 2017). Consistent with previous studies, ChR2-EYFP was intensely expressed in both excitatory and inhibitory neurons in the OB under the promoter human synapsin 1 (hSyn; Kügler et al., 2003; Dittgen et al., 2004; Nathanson et al., 2009; Figure 1B). Heavy expression of EYFP was confined to the superficial portion of layer I in the LEC (Figure 1C), congruent with previous anatomical findings of OB projections (Kosel et al., 1981; Wouterlood and Nederlof, 1983; Room et al., 1984; Wouterlood et al., 1985). We observed somatic labeling in neither superficial nor deep layers in the LEC (Figure 1C), indicating little retrograde transfection of LEC output neurons with axon projections to the OB (Shipley and Adamek, 1984). Therefore, our optogenetic approach enabled us to selectively trace and label direct OB MC axonal projections to LEC.

### OB Input Elicits Excitatory Monosynaptic Responses in LEC Fan Cells

**Figure 2:**
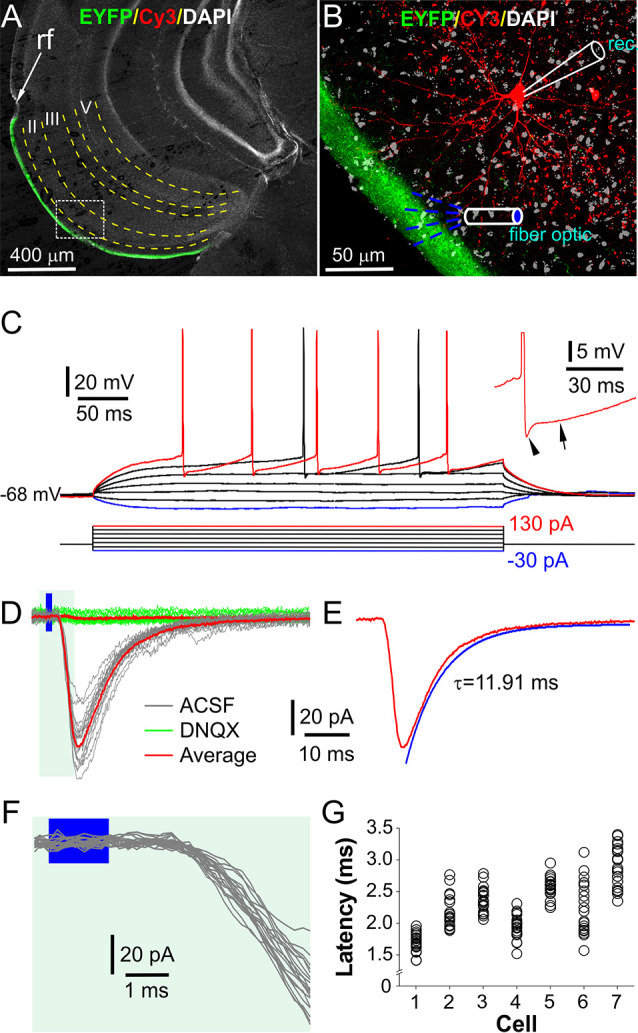
Optical stimulation of layer I elicits excitatorypostsynaptic currents (EPSCs) in fan cells in LEC layer II. **(A)** A typical confocal photo of a brain slice showing EYFP expression (green) in layer I and a biocytin/Cy3-labeled fan cell (FC, red) recorded in layer II. **(B)** A blown-up image of the white box area shown in **(A)** showing the recorded FC, layer I EYFP expression, and recording and stimulation setup. Rec: recording electrode. **(C)** Injected current-evoked voltage (IV) responses of the FC shown in **(A,B)**. Inset highlights the fast and medium hyperpolarization following an action potential (AP). **(D)** Representative voltage-clamp traces showing the FC responses to optical stimulation of layer I as illustrated in **(B)** in the absence (ACSF, gray) or presence (green) of 10 μM DNQX. **(E)** The average trace in ACSF in **(D)** showing the EPSC decay time constant. **(F)** Blown-up traces from **(D)** showing the consistent short onset latencies of the optical stimulation-evoked EPSCs. **(G)** Plot showing the onset latencies of EPSCs recorded in seven FCs (20 traces/cell).

To examine LEC neuron responses to OB input, we focused on layer II neurons (Figure 2A) that have dendrites extending into the LEC layer I and provide axonal projections to the dentate gyrus of the hippocampus (Tahvildari and Alonso, 2005; Canto et al., 2008; Canto and Witter, 2012). To visualize the recorded cells during recording followed by *post hoc* reconstruction, 10 μM Alexa-594 and 0.2% biocytin were included in the pipette solution. The morphological reconstruction showed that 12 out of 20 recorded cells in 20 slices from six animals were fan cells (FCs), the most abundant principal neurons in LEC layer II. The distinctive morphology of these FCs includes: (1) a polygonal soma with multiple thick primary dendrites radiating in the ascending and horizontal directions; and (2) the primary dendrites branching repeatedly to form a semicircular (fan) shape of territory coverage with the base approximately in parallel to layer II (Figure 2B). The average resting membrane potential, input resistance, and action potential (AP) threshold in seven FCs were −73.4 ± 1.5 mV, 224.9 ± 36.3 MΩ, and −43.6 ± 2.1 mV, respectively (Figure 2C). APs were typically followed by fast and medium afterhyperpolarization (Figure 2C inset). These morphological and electrophysiological characteristics were consistent with LEC layer II fan cells in rats (Tahvildari and Alonso, 2005; Canto and Witter, 2012).

To measure OB input-evoked synaptic responses, FCs were voltage-clamped at −70 mV while optical stimulation was presented to layer I with a fiber optic or an objective lens to selectively activate axon fibers projected from the OB (Figure 2B). In these conditions, all FCs responded with inward currents, which exhibited consistently short latencies (2.27 ± 0.15 ms, *n* = 7) with a synaptic jitter of 242.1 ± 36.7 μs (Figures 2F,G), consistent with the monosynaptic transmission (Sabatini and Regehr, 1999; Doyle and Andresen, 2001). These inward currents were completely abolished by bath-applied 10 μM DNQX (Figure 2D), a selective AMPA receptor blocker, suggesting that they are glutamate-mediated excitatory postsynaptic currents (EPSCs). The average peak time and decay time constant of these EPSCs in seven FCs were 4.3 ± 0.4 ms and 11.3 ± 1.7 ms (Figure 2E), respectively. Similar postsynaptic responses were observed in other neurons in layer II of LEC including pyramid cells (*n* = 3), multiform neurons (*n* = 3), and interneurons (*n* = 2). In sum, our results demonstrated an AMPA receptor-mediated monosynaptic transmission from OB axon terminals to FCs in the LEC.

### Dopamine Suppresses EPSCs in Fan Cells

**Figure 3:**
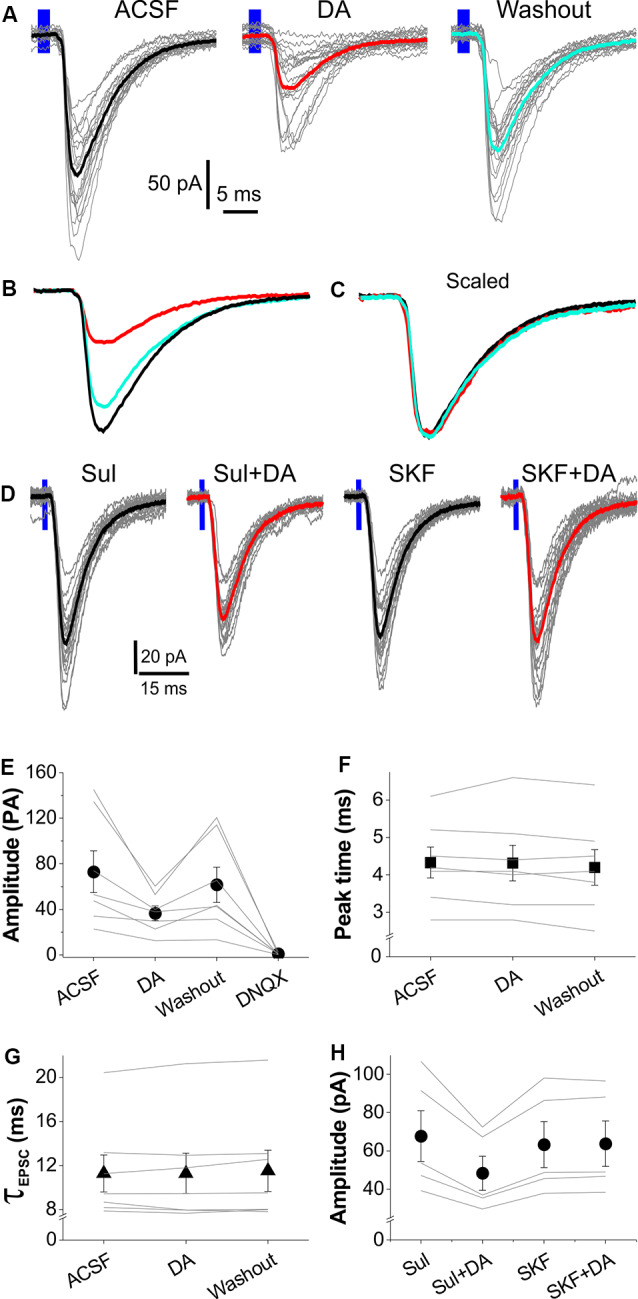
Dopamine (DA) suppresses OB input-evoked EPSCs in LEC layer II FCs. **(A)** Typical voltage-clamp traces showing that optical stimulation of layer I-evoked EPSCs in ACSF, DA, or after DA washout. Thick traces represent the average of each condition. **(B)** Comparison of the average traces from **(A)** showing that DA reversibly reduces EPSCs. **(C)** Scaled average traces showing that DA does not alter the kinetic profile of EPSCs. **(D)** Typical voltage-clamp traces showing that optical stimulation of layer I-evoked EPSCs in the presence of 50 μM (S)-(-)-Sulpiride (Sul), Sul plus 5 μM DA (Sul+DA), 10 μM SKF83566 (SKF) after washout of Sul and DA, and SKF plus 5 μM DA (SKF+DA). **(E)** A graph showing the effects of DA and DNQX on EPSC amplitude in seven FCs. **(F,G)** Plots showing that DA affects neither peak time **(E)** nor decay time constant (τ) of the EPSCs in seven FCs evoked by optical stimulation of layer I. **(H)** Plot showing the effects of 50 μM (S)-(-)-Sulpiride (sul) or 10 μM SKF83566 (SKF) on the DA modulation of amplitude of the optical stimulation-evoked EPSCs in five FCs.

DA has been shown to facilitate or inhibit glutamatergic synaptic transmission in layer II neurons in the LEC including FCs at a low dose (1–10 μM) to activate postsynaptic D1 receptors or at high dose (20–100 μM) to activate presynaptic D2 receptors, respectively (Caruana et al., 2006; Caruana and Chapman, 2008; Glovaci and Chapman, 2015). Considering that a low dose is more physiologically relevant, we tested the effects of DA only at low concentrations (5 μM) on the OB-LEC pathway selectively activated with the optogenetic approach. As shown by Figures 3A,B, bath applied DA after 5 min of stable recording significantly inhibited the optical stimulation-elicited EPSCs in FCs. This effect was partially reversible after 10 min DA washout. The average EPSC amplitude in 7 FCs was reduced by 50.3% from 77.9 ± 25.3 pA in ACSF to 38.7 ± 8.5 pA (*t*_(18)_ = 3.01, *p* = 0.04542) in DA. After DA washout, the EPSC amplitude was resumed to 61.5 ± 15.6 pA (*p* = 1 compared to in ACSF) but was reduced to 0.9 ± 0.3 pA (*t*_(18)_ = 5.0, *p* = 5.54 × 10^−4^ compared to DA washout) in the presence of 10 μM DNQX (Figure 3E), demonstrating a DA suppression of excitatory synaptic transmission from OB input to FCs. However, the kinetic profile of EPSCs was not affected by DA (Figures 3C,F,G). The average peak time and decay time constant were 4.3 ± 0.4 ms and 11.3 ± 1.7 ms in ACSF, 4.3 ± 0.5 ms (*t*_(12)_ = 0.18, *p* = 1 compared to in ACSF) and 11.3 ± 1.8 ms (*t*_(12)_ = 0.05, *p* = 1 compared to in ACSF) in DA, 4.2 ± 0.5 ms (*t*_(12)_ = 1.4, *p* = 0.5547 compared to in DA) and 11.5 ± 1.9 ms (*t*_(12)_ = 1.06, *p* = 0.92941 compared to in DA) after DA washout, respectively (Figures 3F,G). Also, DA altered neither the resting membrane potential nor input resistance of FCs with average values as 73.4 ± 1.5 mV and 224.9 ± 36.3 MΩ in ACSF, 72.8 ± 1.0 mV (*t*_(6)_ = −0.4883, *p* = 0.64267) and 217.4 ± 17.3 MΩ (*t*_(6)_ = −0.0354, *p* = 0.97291) in DA, respectively.

DA inhibits presynaptic release of glutamate at the synapse from cortical input to FCs in the LEC *via* dopamine D2 receptors (Caruana et al., 2006; Caruana and Chapman, 2008), which are intensely expressed in layer I of EC (Charuchinda et al., 1987; Köhler et al., 1991). Thus, we tested whether the inhibitory DA effects on the OB-LEC transmission were mediated by D2 receptors in another set of experiments. Slices were treated with 50 μM (S)-(-)-Sulpiride, a selective DA D2 receptor antagonist (Beaulieu and Gainetdinov, 2011), for 10 min before DA application. In these conditions, bath-applied DA still reduced amplitude of the optical stimulation-evoked EPSCs in five FCs in five slices from two animals (Figures 3D,H). After (S)-(-)-Sulpiride and DA washout, slices were treated with 10 μM SKF83566, a selective D1 receptor antagonist (Beaulieu and Gainetdinov, 2011), for 10 min before addition of DA. In this context, DA exhibited no effect on the OB-FC EPSCs (Figures 3D,H). Input resistance and resting membrane potential of the recorded FCs were altered by neither (S)-(-)-Sulpiride nor SKF83566. Taken together, our results revealed that the DA inhibits the OB-FC synaptic pathway *via* activation of D1 receptors.

### Dopamine Alters Short-Term Plasticity of the OB-LEC Transmission

**Figure 4:**
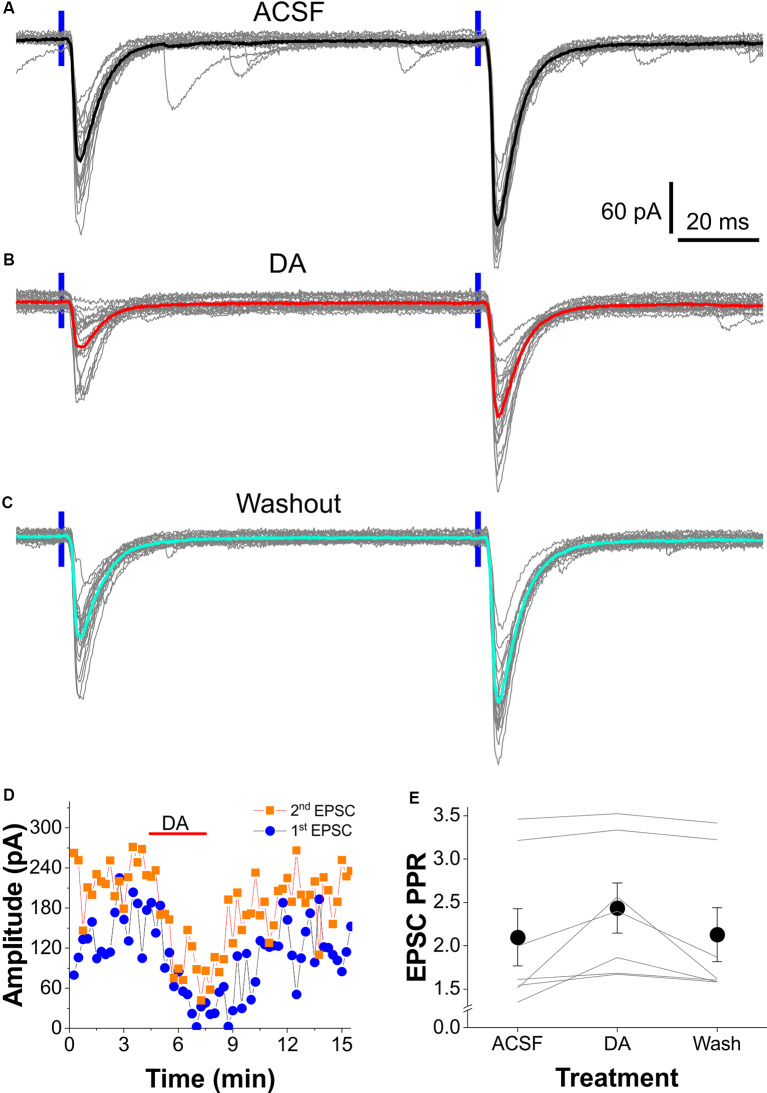
DA alters the paired-pulse facilitation (PPF) of the OB-input-FC synapses. **(A–C)** Typical voltage-clamp traces showing the PPF of EPSCs in an FC evoked by two consecutive optical stimuli (100 ms interval) presented to layer I in ACSF **(A)**, in the presence of 5 μM DA **(B)**, or after DA washout **(C)**. **(D)** Plot of both the first and second EPSC amplitude against time showing the DA suppressive effect in the same cell shown in **(A–C)**. **(E)** Data from a group of seven cells showing that DA reversibly enhances PPF of the OB input-FC synapse.

Paired-pulse ratio (PPR) of synaptic responses is a measurement of short-term synaptic plasticity (Zucker, 1989; Zucker and Regehr, 2002; Jackman and Regehr, 2017). It generally reflects the amount of neurotransmitter released from the presynaptic terminals in response to two stimuli paired at a certain temporal interval. Thus, PPR has been widely employed to assess whether modulation of synaptic transmission originates presynaptically or postsynaptically based on an assumption that, if only postsynaptic mechanisms are involved, both postsynaptic responses should be proportionally affected thus PPR remains unchanged. To determine whether DA reduces synaptic responses to OB input in FCs through presynaptic mechanisms, we recorded and compared FC responses to a paired-pulse optical stimulation of the OB axon fibers in layer I with or without DA in the bath. As shown by Figure 4A, paired-pulse stimuli with an interval at 100 ms elicited robust paired-pulse facilitation (PPF) of EPSCs in FCs. Bath-applied DA significantly reduced the amplitude of both EPSCs (Figures 4B,D) and this action was partially recovered after 10 min of DA washout (Figures 4C,D). The PPR of EPSCs (EPSC2/EPSC1) in all seven FCs was consistently increased by DA (Figure 4E). The average PPR was 2.10 ± 0.33 in ACSF, 2.43 ± 0.29 (*t*_(12)_ = 3.088, *p* = 0.0282 compared to in ACSF) in DA, and 2.13 ± 0.31 (*t*_(12)_ = 0.271, *p* = 1 compared to in ACSF; *t*_(12)_ = 2.817, *p* = 0.04665 compared to in DA) after DA washout. These results support presynaptic inhibition of glutamate release from OB axon terminals by DA onto the LEC FCs.

## Discussion

We for the first time characterized the direct OB input-evoked synaptic responses in FCs, the major principal neuron type in layer II of the LEC, with the optogenetic approach and examined DA modulatory effects with three major findings. First, OB input elicited monosynaptic excitatory responses in all recorded FCs. Second, these synaptic responses exhibit consistently robust PPF and are suppressed by DA *via* D1 receptors. Finally, this inhibitory DA modulation of the OB-FC pathway is due to reduced glutamate release from presynaptic axon terminals as DA increases the PPF of these synaptic responses.

The finding of the OB input-evoked AMPA receptor-mediated monosynaptic excitatory responses in FCs in layer II of LEC in the present study provides the first functional evidence at the single-cell level supporting previous anatomical and electrophysiological findings that MC axons project directly to the LEC (Heimer, 1968; Haberly and Price, 1977; Wouterlood and Nederlof, 1983; Boeijinga and Van Groen, 1984; Wouterlood et al., 1985; Van Groen et al., 1987; Biella and de Curtis, 2000). This is also in agreement with what Caruana et al. (2006) observed in rat LEC FCs, which responded to electrical stimulation of layer I with glutamate-mediated excitatory postsynaptic potentials (EPSPs; Caruana et al., 2006; Caruana and Chapman, 2008). Furthermore, PPF was consistently observed in the layer I-evoked excitatory postsynaptic responses in FCs in studies of and ours (Caruana et al., 2006; Caruana and Chapman, 2008). However, there are multiple distinctions of DA effects on synaptic responses in FCs between studies of Caruana et al. (2006) and ours. First, we found that 5 μM DA consistently suppressed the OB input-evoked EPSCs in FCs while Caruana et al. (2006) observed facilitating effect of DA at low concentration (<10 μM) on the layer I stimulation-evoked EPSPs in FCs. Second, D1 receptors mediate the inhibitory effect DA in our study but the facilitating effect in the studies of Caruana et al. (2006). At least two major factors could contribute to these discrepancies: (1) different stimulation approaches, i.e., we applied optogenetic stimulation to selectively activate axon fibers projected from the OB while in the studies of Caruana et al. (2006) layer I was stimulated by electrical stimulation that activates not only afferent fibers from other cortical and subcortical regions in addition to the OB (Canto et al., 2008) but also potentially local neurons in the superficial layers of LEC that have dendrites extending into layer I (Tahvildari and Alonso, 2005; Canto and Witter, 2012); and (2) the differences in species used, i.e., our subjects were mice while rats were used in the studies of Caruana et al. (2006).

Activation of D1 receptors generally produces excitatory actions in neurons while D2 receptors mediate inhibitory effects (Beaulieu and Gainetdinov, 2011). For example, in the OB activation of D1 receptors in external tufted cells produces an excitatory rebound response while activation of D2 receptors on olfactory nerve terminals inhibits glutamate release (Ennis et al., 2001; Liu et al., 2013; McGann, 2013). Thus, the D1 receptor-mediated DA facilitating effect on the layer I-evoked synaptic responses in FCs in the studies of Caruana et al. (2006); Caruana and Chapman (2008) might be due to activation of D1 receptors on presynaptic axon terminals from origins other than OB. How does activation of D1 receptors produce an inhibitory effect on the OB input-elicited EPSCs in FCs as we observed? Multiple lines of evidence from our study suggest that the inhibitory DA action on the OB-FC synapse is mediated by presynaptic mechanisms. First, DA at the dose we used alters neither resting membrane potential nor input resistance of FCs. Second, the kinetic profiles of the OB-FC EPSCs are not affected by DA, consistent with the insufficiency of activation of DA receptors to modify the number or conductance of synaptic APMA receptors (Tritsch and Sabatini, 2012). Third, DA increases PPF of EPSCs, indicating a reduction of neurotransmitter release from presynaptic terminals. One plausible interpretation is that activation D1 receptor inhibits N-type calcium channels (Kisilevsky et al., 2008) to reduce glutamate release from MC axon terminals in layer I thus suppress postsynaptic responses in FCs as both D1 receptors and N-type calcium channels are expressed in MCs (Coronas et al., 1997; Yuan et al., 2004). Alternatively, DA might activate D1 receptors on other local neuron types, which release adenosine to retrogradely inhibit glutamate release from presynaptic axon terminals of OB MCs in the layer I (Wang et al., 2013) *via* A1 receptors, which are intensely expressed in EC presynaptically (Wardas, 2002). These mechanistic speculations warrant future work.

## Data Availability Statement

All datasets generated for this study are included in the article.

## Ethics Statement

The animal study was reviewed and approved by the Howard University Animal Care and Use Committees.

## Author Contributions

SL planned and performed the experiments, analyzed the data, and performed the statistical analysis, interpreted the results, wrote the manuscript, and approved the content.

## Conflict of Interest

The author declares that the research was conducted in the absence of any commercial or financial relationships that could be construed as a potential conflict of interest.
